# From Diabetes to Atherosclerosis: Potential of Metformin for Management of Cardiovascular Disease

**DOI:** 10.3390/ijms23179738

**Published:** 2022-08-27

**Authors:** Anastasia V. Poznyak, Larisa Litvinova, Paolo Poggio, Donato Moschetta, Vasily Nikolaevich Sukhorukov, Alexander N. Orekhov

**Affiliations:** 1 Institute for Atherosclerosis Research, Osennyaya 4-1-207, 121609 Moscow, Russia; 2 Center for Immunology and Cellular Biotechnology, Immanuel Kant Baltic Federal University, 6 Gaidara Street, 236001 Kaliningrad, Russia; 3 Unit for Study of Aortic, Valvular and Coronary Pathologies, Centro Cardiologico Monzino IRCCS Via Carlo Parea 4, 20138 Milan, Italy; 4 Department of Pharmacological and Biomolecular Sciences, The University of Milan, 20133 Milan, Italy; 5 Laboratory of Angiopathology, Institute of General Pathology and Pathophysiology, 8 Baltiiskaya Street, 125315 Moscow, Russia; 6 Petrovsky National Research Centre of Surgery, 2, Abrikosovsky Lane, 119991 Moscow, Russia

**Keywords:** atherosclerosis, cardiovascular disease, lipid, metformin

## Abstract

Atherosclerosis is a common cause of cardiovascular disease, which, in turn, is often fatal. Today, we know a lot about the pathogenesis of atherosclerosis. However, the main knowledge is that the disease is extremely complicated. The development of atherosclerosis is associated with more than one molecular mechanism, each making a significant contribution. These mechanisms include endothelial dysfunction, inflammation, mitochondrial dysfunction, oxidative stress, and lipid metabolism disorders. This complexity inevitably leads to difficulties in treatment and prevention. One of the possible therapeutic options for atherosclerosis and its consequences may be metformin, which has already proven itself in the treatment of diabetes. Both diabetes and atherosclerosis are complex metabolic diseases, the pathogenesis of which involves many different mechanisms, including those common to both diseases. This makes metformin a suitable candidate for investigating its efficacy in cardiovascular disease. In this review, we highlight aspects such as the mechanisms of action and targets of metformin, in addition to summarizing the available data from clinical trials on the effective reduction of cardiovascular risks.

## 1. Introduction

Originating from the Greek language, the term atherosclerosis literally means “porridge or grain”, since this is what the lipid material located in the core of the typical atheroma (or atherosclerotic plaque) looks like [1]. In atherosclerosis, fatty and/or fibrous material is accumulated in the intima, which is the innermost layer of the arteries. After a while, the atheroma becomes more fibrous and stores calcium [2,3].

Advanced atherosclerotic plaques can penetrate the lumen of the arteries, thereby interfering with blood circulation and resulting in tissue ischemia. As for plaques that do not create obstacles, hindering the flow can cause blood clot formation, which, in turn, can clog the lumen, thus giving a second and more acute path to ischemia [4]. As before, atherosclerotic cardiovascular diseases (CVD) is globally the most frequent key cause of vascular disease these days. When CVD affects the heart’s blood circulation, it potentially results in severe coronary syndromes such as myocardial infarction or chronic conditions, e.g., stable angina pectoris (chest pain or malaise due to unsatisfactory perfusion of the heart muscle) [5]. The majority of cases of ischemic stroke and transient ischemic attacks of the brain—which may result in the appearance of aneurysms, including those formed in the abdominal aorta—occur because of the contribution of atherosclerosis. Lesions of the peripheral arteries may cause periodic lameness, ulceration, and gangrene, which can put the viability of the limbs at risk [6,7,8].

Today, we know quite a lot about atherosclerosis from a clinical point of view. We are well aware of the danger of this disease and its potential consequences. The pathogenesis of this disease has also been well investigated. It is well known how atherosclerosis develops, and many molecular participants in this process and their relationships with each other have been identified. However, despite the abundance of diverse information and a large number of studies on atherogenesis, these data are not always linked with each other and, as a result, do not allow us to form a complete picture. One of the crucial gaps in our knowledge is the trigger mechanism for atherosclerosis. There are several different versions on what is the starting point, but none of them have been confirmed yet.

**Figure 1:**
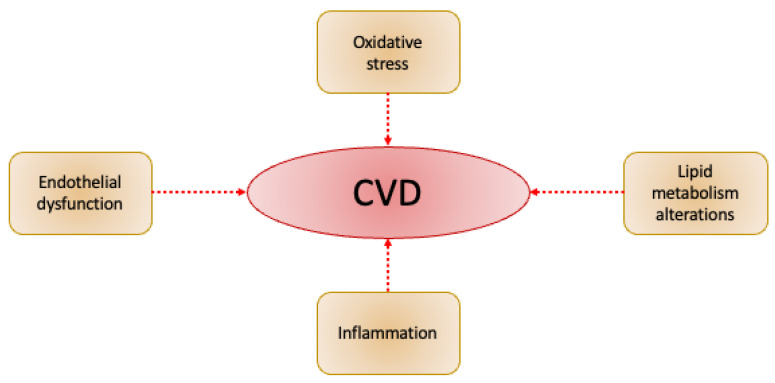
Scheme of essential components of CVD.

Most likely, it is this gap in our knowledge about atherosclerosis that is reflected in the problems of treatment and prevention. To date, there is no absolutely effective treatment or universal prevention of atherosclerosis. Many drugs are being tested for their effectiveness in treating atherosclerosis. Among them, priority is given to those that have already proven themselves in the treatment of diseases associated with one of the key mechanisms involved in atherogenesis: inflammation, oxidative stress, and lipid metabolism disorders (see Figure 1). One such drug is metformin. It has proven itself in type 2 diabetes therapy, which is the most common disease associated with impaired lipid metabolism. Given the many similarities in the processes underlying these two diseases, the question naturally arises, can a drug that is effective in one case be effective in the other?

## 2. Metformin

For almost a century, the most widespread drug for the therapy of type 2 diabetes (T2D) was metformin, the derivative of biguanide. In 1918, it was found that guanidine has antidiabetic properties in animals, but to everyone’s disappointment, in clinical trials, it appeared to be toxic [9]. This inspired scientists to find safer replacements. Metformin (1,1-dimethyl biguanide hydrochloride) was synthesized in the 1920s. Ever since, it has become a priority drug for T2D treatment due to its ability to decrease plasma glucose levels [10]. Over the last few years, many other beneficial functions of metformin have been revealed. The results reported that metformin strongly affects numerous types of cancer, CVD, liver disease, obesity, neurodegenerative disease, and kidney disease. The use of a single drug or conjunction therapy with other agents has demonstrated its effectiveness for the treatment of various diseases [11].

Metformin suppresses mitochondrial complex I, which leads to activation of adenosine 5′-monophosphate-activated protein kinase (AMPK). Mitochondrial complex I is crucial for electron transfer. Consequently, the generation of adenosine triphosphate (ATP) lowers, and the intracellular concentration of adenosine diphosphate (ADP) elevates. Therefore, the adenosine monophosphate (AMP) cellular levels increase, which ultimately activates AMPK [12,13,14]. Furthermore, a recent study has demonstrated that metformin can activate APK via the lysosomal pathway, i.e., the AX-IN/LKB1-v-ATPase-Regulator pathway. AMPS is a key regulator for almost all metabolic pathways, including glucose metabolism, lipid metabolism, and energy homeostasis [15]. Moreover, by transmitting insulin signals and IGF receptors, which result in metabolic homeostasis changes, metformin plays an important role. Using the hdPCA strategy (“homomer dynamics’’ protein fragment complementation assays), which can show gene functions and drug target pathways, it was discovered that the protein levels responsible for a wide range of cellular processes (including energy metabolism, aging, and cancer) were altered by metformin [16]. Despite this, the main mechanisms of metformin in the regulation of diseases have yet to be fully studied.

## 3. Diabetes

Various pieces of research and clinical trials have shown that metformin monotherapy or conjunction therapy with other hypoglycemic drugs has good results in the treatment of T2D. In 1995, a report demonstrated that metformin could decrease plasma glucose levels [17]. A study conducted by Defronzo et al. in 1995 [18] involved 289 patients suffering from diabetes who took either metformin or placebo. Twenty-nine weeks later, lower average fasting plasma glucose and HbA1c levels were observed in the group of patients receiving metformin. Another study was conducted by Garber in 1997 [19], enrolling 451 diabetic patients who received various daily doses of metformin (from 500 mg to 2000 mg per day). Fourteen weeks later, the effectiveness of metformin was found to depend directly on the dose. In 2006, the results of a randomized, double-blind, five-year clinical trial were published. In this study, metformin was compared with other antidiabetic drugs such as glibenclamide and rosiglitazone. This study’s outcome demonstrated that fasting plasma glucose levels were reduced to a lesser extent by rosiglitazone and to a greater extent by glibenclamide; in addition, metformin also showed intermediate effects [20].

Under some circumstances, metformin is used with other antidiabetic drugs. For instance, a study lasting 29 weeks, in which 632 people participated, showed that the conjunction of metformin and glibenclamide gives better glucose control than metformin alone [18]. In a clinical trial involving 372 people, glimepiride demonstrated similar results. Another study demonstrated that after 3 months of treatment, metformin and troglitazone contribute to a more powerful decrease in plasma glucose levels on an empty stomach and after meals, in contrast to treatment with metformin alone [21]. In addition, studies have made it clear that conjunction therapy of metformin with DPP 4 suppressors, SGLT2 suppressors, or GLP 1 receptor agonists demonstrated successful glucose control without the extra risk of hypoglycemia [22]. Another effective way to treat diabetes is a combination of metformin and insulin. The use of this combination in a study among 96 patients gave an understanding that the conjunction of drugs gives better glucose control and weight gain, unlike treatment with metformin alone [23,24]. Another study involving 390 patients showed that, in contrast to therapy with metformin alone, the use of conjunction with insulin also demonstrated the best control of glucose levels [25].

In patients suffering from cognitive disorder and glucose metabolism disorder, metformin enhances insulin susceptibility and lowers fasting insulin levels. For pregnant women who suffer from type 2 diabetes, gestational diabetes (GDM), and polycystic ovary syndrome (PCOS), the use of metformin is the most appropriate treatment solution [26]. Because of in vitro and in vivo studies, which also include clinical trials and animal studies, metformin therapy for pregnant women is becoming increasingly popular worldwide. However, the safety of using this drug during pregnancy is controversial [27]. Studies have demonstrated that children whose mothers used metformin during pregnancy may subsequently have a stronger tendency to BMI, abdominal fat volume, or blood pressure [28]. Studies among patients who received metformin for more than 10 years have shown that these patients had an elevated risk of beta-cell insufficiency and insulin resistance [29]. Even though long-term follow-up studies may be necessary to establish the potential effects of metformin on human cells and tissues, metformin certainly remains the most appropriate treatment strategy for people with diabetes.

## 4. Metformin Action Mechanisms

**Figure 2:**
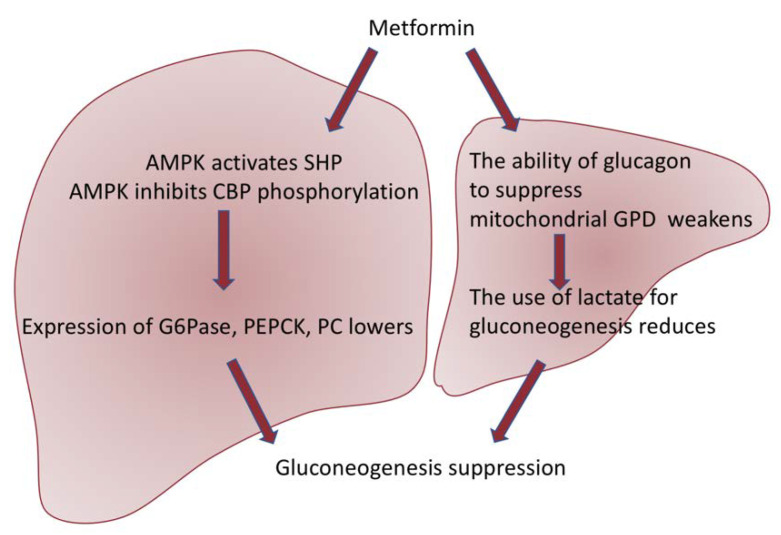
Scheme of two pathways of metformin action. Metformin suppresses gluconeogenesis through AMPK-dependent activation of SHP (small heterodimeric partner) and suppression of phosphorylation of CBP (CREB-binding protein), thus inhibiting the expression of gluconeogenic genes such as G6Pase (glucose-6-phosphatase), PEPCK (phosphoenolpyruvate carboxykinase), and PC (pyruvate carboxylase) [32]. In addition, AMPK activation results in the suppression of mTORC1 (the mammalian target of the rapamycin I complex), which also leads to the inhibition of gluconeogenesis [33]. Moreover, metformin suppresses the generation of glucose in the liver in a way independent of AMPK. Studies have revealed that metformin weakens the ability of glucagon to suppress mitochondrial GPD (glycerol-3-phosphate dehydrogenase), which further results in a violation of the use of lactate for gluconeogenesis [34].

Metformin provides antihyperglycemic action mainly by inhibiting glucose production in the liver through AMPK-dependent or independent pathways, which are briefly reflected in Figure 2. One recent study also showed that metformin directly targets FTP 1 (fructose-1,6-bisphosphatase-1), an enzyme that controls the rate of gluconeogenesis, suppressing glucose production in the liver [30]. Other studies have revealed that metformin is also able to strengthen GLUT 1 (glucose transporter 1) mediated by glucose transporter to hepatocytes by activating IRS2 (substrate of the second insulin receptor), lowering plasma glucose levels [31].

In addition to reducing glucose generation by the liver, metformin also lowers glucose levels by elevating GLUT4 (glucose transporter 4), mediated by glucose absorption in skeletal muscles and glucose absorption in the intestine [35]. Metformin also promotes the release of GLP-1 (glucagon-like peptide-1), which increases insulin secretion and decreases plasma glucose levels [36]. Moreover, recent studies have revealed that the gut microbiota can become a target of metformin. Several studies report that gut microbiota dysbiosis observed in T2D patients is still growing. As a result of a randomized, double-blind study, it was established that metformin affects the composition and function of the gut microbiota [37]. This allows us to take a fresh look at the mechanism that underlies the antidiabetic effects of metformin. After short-term administration of metformin, the amount of Bacteroides fragilis in the intestine lowered, which led to an elevation in the level of GUDCA (glycursodexoycholic acid). An increase in the level of GUDKA inhibits intestinal FXR (farnesoid X-receptor), which leads to an enhancement in glucose tolerance [38].

Gluconeogenesis is an energy-intensive process, which creates the necessity for balancing of the ATP consumption regarding the demand of energy in hepatocytes. This is released by mitochondria. Metformin has a positive charge, and the membrane potentials across the plasma membrane and mitochondrial inner membrane (positive outside) drive metformin into the cell and subsequently into the mitochondria. One of the well-known mechanisms of mitochondrial actions of metformin is the inhibition of Complex I of the respiratory chain, which inhibits the generation of ATP [39]. However, these effects received a lot of criticism regarding the high extracellular concentrations (mmol/L) required to observe rapid effects, although lower concentrations of metformin (50–100 μmol/L) do inhibit Complex I in rat hepatoma (H4IIE) cells after several hours. This delay can be explained by the slow uptake of metformin by mitochondria. Moreover, no changes in cellular ADP:ATP ratios after metformin treatment were detected, but they were observed after the use of phenformin. The inhibition of this pathway in cells responsible for gluconeogenesis can, at least in part, explain modest effects on ADP:ATP ratios. Furthermore, apart from the ATP generation, other alterations of the respiratory chain (e.g., the NAD+:NADH ratio) may be related to the metformin effects on gluconeogenesis [40].

An alternative target of metformin in mitochondria was proposed recently. This could be glycerophosphate shuttle, which is the system that carries reducing equivalents from the cytoplasm into the mitochondrion for re-oxidation. An essential component of this shuttle, mitochondrial glycerophosphate dehydrogenase (mGPD), appeared to be inhibited by metformin in cell-free analysis. However, this inhibition can be insufficient for a sustained impact on gluconeogenesis because the malate/aspartate shuttle will compensate unless the mitochondrial membrane potential (which is maintained by the respiratory chain) also becomes suppressed [41].

### 4.1. Metformin Affects Muscles

Apart from suppressing gluconeogenesis and HGP in the liver, metformin affects various organs and tissues. Thus, metformin was shown to increase insulin-stimulated glucose uptake in skeletal muscle. It was shown that the activity of AMPK and phosphorylation was enhanced in muscle biopsies from patients with T2D following metformin treatment. According to these findings, Inzucchi et al. revealed that insulin-stimulated peripheral glucose uptake increased by 13%, along with ~20% reduction in rates of HGP, resulting in a 58 mg/dL reduction in fasting plasma glucose concentration in patients with poorly controlled T2D after 3 months of metformin (1000 mg twice a day) treatment [42]. However, it seems that such an effect of metformin to stimulate enhanced insulin-stimulated peripheral glucose uptake is an indirect effect related to reductions in glucose toxicity. Consistent with this suggestion, no effects of metformin were shown in the study of Yu et al. They observed the effects of insulin-stimulated peripheral glucose metabolism on T2D patients rendered normoglycemic following 4 weeks of continuous subcutaneous insulin, who received metformin (850 mg twice/day) treatment [43]. These results were then confirmed with positron emission tomography (PET) imaging, which revealed that the observed increase in whole-body glucose uptake could be dissociated from skeletal muscle-specific effects [44].

### 4.2. Metformin Affects Intestines

Intestinal mechanisms of metformin effects have been recognized, along with the gastrointestinal side effects that are observed in some patients. Observational studies revealed that 16–62% of patients had gastrointestinal side effects, leading to metformin intolerance in about 5% of patients [45].

Beneficial effects of metformin can be linked to the alterations in gut microbiome composition, intestinal glucose uptake, and hormone (e.g., growth differentiation factor 15 (GDF15), glucagon-like peptide-1 (GLP-1)) secretion. The alteration of gut microbiome was shown in T2D patients, but the association with the glucose-lowering effects of metformin is doubtful. Results obtained in a murine model support this suggestion, but more convincing data is needed [46].

Another intestinal effect of metformin action is related to GDF15 secretion. Elevated serum GDF15 was shown in patients with T2D, and this was associated with the treatment with metformin. The potential mechanism lies in the fact that metformin-induced activation of the integrated stress response pathway leads to GDF15 secretion, which improves glycemic regulation and reduces appetite. It is important to note that in vitro, an enhanced expression of GDF15 as well as enhanced secretion from hepatocytes was shown [47].

Among additional mechanisms of metformin affecting intestines, there are enhanced GLP-1 secretion, delayed gastric emptying, and altered enterocyte glucose metabolism. Different investigations revealed a direct effect on GLP-1 expression, indirect effects through dipeptidyl peptidase-4 (DPP4) activity, or no effect on GLP-1 at all [48].

### 4.3. Metformin Affects Adipose Tissue

In adipose tissue, anti-inflammatory cytokines are expressed by a network of type 2 immune cells. These cytokines stimulate macrophage polarization, leading to their transition to an alternatively activated ‘M2-like’ state. These M2-like macrophages favor the resolution of inflammation and tissue-specific insulin responsiveness, with the latter through a currently undefined mechanism [49]. In obesity and T2D, this system starts to work incorrectly. This leads to the change in adipose tissue macrophage polarization in favor of classically activated M1-like macrophages, which orchestrate insulin resistance through enhanced secretion of pro-inflammatory cytokines [50].

## 5. Cardiovascular Disease

Currently, the most frequent cause of death and disability is cardiovascular disease (CVD). There are many prerequisites for CVD development, including, for example, smoking, diabetes, obesity, hyperlipemia, and hypertension. Often, in conjunction with CVD, both T1D and T2D can be found [51]. Hyperglycemia causes oxidative stress, thus resulting in endothelial dysfunction, which elevates the risk of CVD development. It was found that the widely used antihyperglycemic drug metformin leads to a decrease in the frequency of CVD in patients with diabetes mellitus [52]. When activating AMPK, metformin suppresses alpha-dicarbonyl-mediated modification of apolipoprotein residues, thereby improving the dysfunction of HDL and decreasing modifications of LDL. Lowering HDL dysfunction enhances cholesterol transport and diminishes cardiovascular risk. In addition, metformin enhances the level of oxidative stress of the endothelium and weakens inflammation caused by hyperglycemia, reducing the incidence of cardiovascular diseases [53,54].

T2D is also related to a higher incidence of heart failure. Studies have reported that almost a third of heart failure cases occur in patients with diabetes. It was determined that by improving the cellular metabolism of lipids and glucose with AMPK, metformin improves the energy status of the myocardium [55]. One of the recent randomized, controlled trials conducted among patients with CHD without diabetes showed that metformin significantly decreased left ventricular hypertrophy (LVH), which is a key prognostic factor in CVD. This study showed that metformin significantly reduces the mass of the left ventricle, indexed by height, left ventricular mass, body weight, and oxidative stress [56]. Reports from other studies revealed the advantages of metformin in cardiovascular diseases and heart failure for both diabetic and non-diabetic patients. Therefore, the study of further metformin use is of particular interest [57].

One of the key stages of atherosclerosis is the absorption of lipids, including oxidized LDL (Ox-LDL) by macrophages and their further apoptosis [58,59]. There is direct evidence that metformin can suppress both of these processes. In human THP-1 cells, a monocyte-like cell line, it has been shown that metformin can weaken an absorption of Ox-LDL by lowering the expression of scavenger receptors (SRA and CD36) through inhibiting b-catenin and activation of protein-1 and PPAR-g. Also, it can weaken Ox-LDL-induced endoplasmic reticulum stress and the formation of reactive oxygen species, as well as Ox-LDL-induced mitochondrial membrane depolarization and cytokine release. In the same cell line, an independent group showed that metformin induces AMPK activation, which suppresses cholesterol uptake mediated by sterol regulatory element-binding protein 2 (SREBP2) [60].

THP-1 cells and macrophages derived from type 2 diabetes patients cultured with high glucose were used for an experiment in which metformin activated AMPK. The mechanism involved the decrease of the cyclophilin A expression (pro-inflammatory), inhibited scavenger receptors and lipid absorption, suppressed foam cell formation, the toxicity of reactive oxygen species, and the release of inflammatory cytokines [61]. The importance of AMPK activation by metformin was also assessed in other vascular cells. In cultured smooth muscle cells of the human aorta, metformin-activated AMP upregulates p53, and IF116 suppresses cell proliferation and migration. In cultured (early passage) human aortic endothelial cells, metformin activates AMPKa and induces telomere expansion of hTERT, delaying cell aging [62]. Prolonged metformin administration lowered mitochondrial biogenesis, which depended on the methylation of H3K79 in the SIRT3 promoter. In additional tests on APOE-/- mice, vascular aging and plaque formation were reduced due to metformin [63,64,65]. The conclusions of human research with metformin have been summed up. Recent studies provide new knowledge about the cardiometabolic effects of the old drug, which underscores the need for further studies of cardiovascular outcomes in people with T1M and non-diabetic dysglycemia [66]. On the other hand, molecular and cellular studies provide clarity about the action mechanisms of metformin on the vascular network, offering new biomarkers that still need to be tested in independent groups. These studies can guide the development and targeting of new agents for the treatment and prevention of CVD and metabolic diseases [55].

## 6. Clinical Trials

Coronary Artery Disease (CAD) is a significant worldwide burden for both health and the economy, as it is related to high morbidity and mortality. It is for this reason that the most important components of patient care are early identification, diagnosis, and initial treatment. Diabetes is a crucial independent risk factor for CAD development. Some clinical studies have demonstrated that in patients with diabetes, metformin reduces cardiovascular events [67].

To determine whether metformin (together with insulin therapy) decreases atherosclerosis in patients with T1DM who have a greater risk of CVD, Petrie et al. performed a double-blind placebo-controlled study (REMOVAL) [68]. The study involved 23 hospital diabetes clinics in 5 different countries: Great Britain, Canada, the Netherlands, Australia, and Denmark. Among the patients were people aged ≥40 years, with T1DM for at least 5 years and at least 10 unique risk factors for CVD; patients were randomized and received either placebo or metformin (1 g twice daily). Among 428 randomly selected people, 219 patients took a placebo and 209 patients took metformin. The carotid intima-media thickness test (CIMT, a surrogate measure of atherosclerosis) showed no decrease in development in the group receiving metformin, while a decrease in the maximum value of CIMT was detected in the same group. Within 3 years, the need for insulin in the group taking metformin significantly decreased. However, body weight and LDL-c (LDL cholesterol levels) were reduced, while the estimated glomerular filtration rate (eGFR) increased. Thus, based on these outcomes, we can conclude that metformin is able to significantly affect the management of CV risks. In children with DM1, vascular dysfunction occurs even before atherosclerosis. To avoid the development of cardiovascular disease, early intervention is necessary. A randomized, controlled, double-blind, 1-year study that involved 90 people demonstrated that metformin at a dosage of 1 g twice a day is capable of improving SMCs and HbA1c function in children with T1DM and reducing insulin doses [69].

Another study was conducted to understand how metformin therapy can affect mortality caused by atherosclerotic thrombosis in diabetic patients. In this study, conducted from 2003 to 2004 as part of the Reduction of Atherosclerosis Continuing Health (REACH), 19,691 patients with diabetes and atherosclerosis took part [70]. Patients either received metformin or did not receive the drug, so it was possible to analyze the two-year mortality rate in these two groups. A two-year mortality rate was analyzed among two groups (patients who used metformin and patients who did not use metformin). The analyses showed that among the group treated with metformin, mortality rates were 6.3%, while among the group not treated with metformin, mortality was 9.8%, and the adjusted hazard ratio (HR) was 0.76 (0.65–0.89; *p* < 0.001). Between these subgroups, the correlation of lower mortality was consistent. Patients who had already been diagnosed with congestive heart failure were in a more advantageous position with a heart rate of 0.69 (0.54–0.90; *p* = 0.006). These outcomes demonstrate that metformin can be used as a secondary prevention measure, lowering mortality in patients with diabetes.

Roumie et al. investigated the cardiovascular clinical results of metformin among patients with T2DM and renal insufficiency [71]. In addition, in patients who had used metformin or sulfonylurea, follow-up began with a reduced threshold of renal function, continuing until the onset of major adverse cardiac events (MACE) (for example, myocardial infarction, heart failure, transient ischemic attack (TIA), ischemic or hemorrhagic stroke, and death from cardiovascular disease), treatment changes, completion of follow-up, death, or completion of the study. The outcomes report that the number of patients who received only metformin or sulfonylureas for a long time was 67,749 and 28,976. In the weighted cohort, there were 24,679 patients receiving metformin and 24,799 receiving sulfonylureas. During the entire follow-up (for metformin, the median is 1.0 years; for sulfonylurea, the median is 1.2 years), 1394 events occurred in users of sulfonylurea (29.2 per thousand person-years) and 1048 MACE events occurred in the metformin group (23.0 per thousand person-years). In contrast to sulfonylureas, the MACE adjusted hazard ratio of metformin is 0.80 (95% CI: 0.75–0.86). This research proves that, unlike sulfonylureas in diabetic patients suffering from renal insufficiency and receiving monotherapy, treatment with metformin may relate to a reduction in the risk of MACE.

Within the framework of the Lawrence et al. study, the effect of an oral hypoglycemic drug on lipoprotein subcomponents in patients with TDM was assessed [72]. Sixty over-weight T2DM patients with no experience of lipid-lowering treatment were arbitrarily separated and prescribed metformin, pioglitazone, or gliclazide after 3 months of a diet, with a change in the dose of medication to optimize blood glucose control and continuation of treatment for 3 months. The content of both HDL and LDL subgroups in the pioglitazone or metformin group changed for the better.

These changes may be linked with a decrease in the risk of atherosclerosis. In addition, a randomized placebo-controlled clinical trial assessed the effect of lifestyle changes (7% weight loss due to a low-fat diet) or metformin (850 mg 2 times per day) on patients with impaired glucose tolerance (IGT). LSM elevates large HDL and reduces small HDL, small and dense LDL, and Very Low-Density Lipoproteins (VLDL). Metformin slightly elevated small and large HDL levels, and also lowered small and dense LDL levels. Metformin therapy had a positive effect on the lipoprotein sub-component, but LSM appears to be more beneficial. Ultimately, these two methods of intervention may hinder atherosclerosis development and progression [73].

Patients with prediabetes (pre-DM) have a more serious risk of developing coronary artery disease; preventive measures may be required to reduce this risk. As part of the Diabetes Prevention Program Outcome Study (DPPOS) and the Diabetes Prevention Program (DPP), 3234 patients with pre-diabetes were studied [74]. After 14 years of observation, measurements of calcium in the coronary arteries (CAC) were carried out to determine the presence of atherosclerosis among 2029 participants. Men in the group of patients taking metformin showed lower severity and the presence of CAC in contrast with the group taking a placebo; however, it is important to note that women did not benefit from metformin. In men with pre-diabetes and early diabetes, metformin (with a dosage of 850 mg twice a day) was able to prevent the occurrence of coronary artery disease. Patients with HIV are often exposed to various metabolic disorders, such as diabetes, obesity, hyperlipidemia, and hypertension. All these disorders can provoke CAC. A study of 50 HIV-infected patients with metabolic syndrome (MetS) was conducted, in which the effect of treatment with LSM and/or metformin (850 mg twice a day) on the parameters of MetS was analyzed [75]. Patients treated with metformin for more than 1 year demonstrated a serious decrease in progression of CAC, while d-Lysergic acid morpholide (LSM) did not affect CAC progression at all. Thus, it becomes clear that treatment with metformin is able to prevent the formation of plaques in HIV-infected patients with MetS.

Endothelial dysfunction is a preliminary characteristic of atherosclerosis in patients with T2DM. Thirty-one volunteers who were related to first-degree patients with DM2 and had normal glucose tolerance and MetS were recruited [76]. The volunteers were randomly divided into two groups: the metformin group (850 mg twice daily) (n = 16) or the control group (n = 15). In the group taking metformin, body weight, BMI, fasting blood glucose (FPG), and systolic blood pressure (BP) decreased, and blood lipids improved. Improvements were also seen in measured endothelium-dependent forearm blood flow (FBF) response. These outcomes demonstrate that metformin can enhance vascular endothelial response in first-degree relatives in patients with T2DM with MetS.

Similarly, among 258 patients with stable angina, in contrast to propensity scores, there were 86 patients with normal blood glucose (NG), 86 pre-DM patients, and 86 persons who received metformin before diabetes (Met + pre-DM). At the end of the second year of observation, the MACE of NG subjects and Met + pre-DM subjects were lower than those of pre-DM subjects [77,78]. The percentage of endothelial dysfunction of the left anterior descending coronary artery (LAD) in patients with NG and Met+ before CT was also lower than in pre-DM patients. These results report that in patients with pre-DM, metformin therapy results in a decrease in the risk of developing MACE via improving the function of the coronary endothelium. In patients with coronary artery disease, insulin resistance (IR), and/or prediabetes, metformin also has a positive effect on the regression of left ventricular hypertrophy (LVH). In a study by Mohan et al., 68 patients with no history of diabetes, but with coronary artery disease, IR, and/or prediabetes, were randomly assigned to 2 groups, one of which was taking metformin (2 g once a day), whilst the second group was taking a placebo for 1 year. The results of this study came down to the fact that metformin therapy reduced body weight, left ventricular mass (LVM), left ventricular mass indexed to height (LVMI), and systolic BP [79]. We summarized the data from clinical trials in the Table 1.

Sodium-glucose cotransporter 2 (SGLT2) inhibitors appeared to be a promising option for therapy in combination with metformin. Agents of this group act in an insulin-independent way, decreasing the blood glucose level via enhancing the urinary excretion of glucose. Also, SGLT2 inhibitors reduce body weight and blood pressure compared with placebo. The range of SGLT2 inhibitors were shown to reduce the risk of hospitalization from heart failure among people with type 2 diabetes with established cardiovascular disease or multiple cardiovascular risk factors [93]. However, Comparison studies that were aimed at assessment of the differences between monotherapy and combined treatment had various limitations and led to controversial results. Thus, the beneficial effects of the use of such a drug combination need to be evaluated.

GLP1 is important for intestinal hormonal pathways, such as incretin effect. GLP1 is deficient in T2DM, but its biological potency is largely retained, making GLP1 an attractive target molecule for therapeutics. Combination treatment with metformin and liraglutide, a GLP-1 analogue, has a synergistic effect against endothelial dysfunction. The function of metformin in upregulating GLP-1R level in endothelial cells potentially contributes to the synergistic protective effect of this combination therapy, which provides new insights into the interaction between metformin and GLP-1 agents. It is conceivable that metformin can potentiate or strengthen the vascular protective effects of GLP-1 and its analogues [94].

EMPA-REG and CANVAS trials are also worth mentioning in this section. These trials were dedicated not to metformin, but SGLT2i. A total of 7020 patients with diagnosed diabetes and CVD were enrolled into the EMPA-REG trial. The participants were divided into two groups: receiving daily empagliflozin at 10 or 25 mg versus placebo. Over a follow-up period of 3.01 years, the trial revealed a modest but significant decrease in the primary outcome of empagliflozin being favored over placebo and a decrease in relative risk reduction and cardiovascular mortality [95].

The CANVAS study reflects the data from two clinical trials on canagliflozin (CANVAS and CANVAS-R). In total, 10,142 patients participated in the CANVAS study (4330 from CANVAS and 5812 from CANVAS-R). The results showed a significant decrease in the primary composite outcome of CV mortality, non-fatal stroke, and myocardial infarctions in the canagliflozin group [96].

DECLARE-TIMI 58 is also a recent trial on dapagliflozin for CV safety/efficacy in a randomized, double-blind, placebo-controlled fashion. This study enrolled 17,160 diabetic patients. The addition of 10 mg of dapagliflozin did not decrease the combined major adverse cardiac events (MACE) outcome, but did show a statistically significant reduction in the co-primary outcome of death or hospitalization for heart failure. The results of these trials reflect the benefits for CVD from the antidiabetic drugs that go beyond the use of metformin [96].

## 7. Conclusions

There is no doubt about the beneficial effect of metformin on the course of diabetes mellitus. Despite all the recent developments, it remains the first drug of choice in prescribing therapy.

As is known, atherosclerosis is also a complex disease; in its pathogenesis, many different molecular mechanisms are involved. In particular, as in the case of diabetes mellitus, this is inflammation and a violation of lipid metabolism.

Taking into account these similarities in the pathogenesis of the two diseases, namely diabetes mellitus and atherosclerosis, it seems quite logical to assume the effectiveness of metformin in the treatment of atherosclerosis. This has been confirmed by numerous clinical studies, which also show that the use of metformin can have a beneficial effect on patients with various diseases of the heart and blood vessels. Data accumulated over decades of scientific research suggest that metformin may be a good component of atherosclerosis therapy. However, based on the available clinical data and taking into account the complexity of the pathogenesis of atherosclerosis, we, like many researchers, consider a strategy that includes the use of a combination of drugs to be more effective. In particular, SGLT2 inhibitors, or GLP 1 receptor agonists, appear to be suitable candidates, but the optimal and most effective combination is yet to be determined.

## Figures and Tables

**Table 1:** Various effects of metformin on endothelial dysfunction.

Subject	Beneficial Effect	Adverse Effect	Mechanism	Reference
Otsuka Long-Evans Tokushima fatty (OLETF) rats (a type 2 diabetes model)	Normalized endothelial function	--	Suppression of vasoconstrictor prostanoids in mesenteric arteries	[80,81,82]
Spontaneously-hypertensive-rats (SHR)	Blood pressure reduction and endothelial-dependent relaxation improvement		Upregulation of NO and, in particular, EDHF	[83]
In vito; streptozotocin diabetes model in vivo	Tissue-intact and cultured vascular endothelial cells protection from hyperglycemia/ROS-induced dysfunction	--	Attenuation of hyperglycemia-induced ROS production in aorta-derived endothelial cell cultures; hyperglycemia-induced endothelial mitochondrial dysfunction prevention (oxygen consumption rate reduction)	[84]
HUVECs; C57/BL6 male mice	Prevention of methylglyoxal-induced apoptosis	--	MGO-induced HUVEC apoptosis prevention; apoptosis-associated biochemical changes inhibition (loss of MMP, the elevation of the Bax/Bcl-2 ratio, and activation of cleaved caspase-3); attenuation of MGO-induced mitochondrial morphological alterations in a dose-dependent manner	[85]
Cultured smooth muscle cells of the human aorta	Delay of cell aging	--	Metformin-activated AMP upregulates p53, and IF116 suppresses cell proliferation and migration. In cultured (early passage) human aortic endothelial cells, metformin activates AMPKa and induces telomere expansion of hTERT, delaying cell aging	[61]
T2D patients with stable coronary heart disease	Lowering of plasma sVCAM-1 (553 ± 148 vs. 668 ± 170 µg/L, *p* = 0.004) and elevation of ADMA (0.53 ± 0.09 vs. 0.48 ± 0.08 µM, *p* = 0.01)	--	Change of VCAM1 and asymmetric dimethylarginine (ADMA) level	[86]
T2D patients treated with insulin	About 34% reduction in the risk of CV morbidity and mortality	--	There was a reduction in the levels of vWF, sVCAM-1, t-PA, PAI-1, CRP, and sICAM-1. No effects on urinary albumin excretion or sE-selectin were observed.	[87,88]
Women with obesity and type 2 diabetes, drug-naïve	Nutritive microvascular reactivity improvement at the capillary level	Unexpected increase in tumor necrosis factor-α	Reduction of weight, plasma glucose, total cholesterol, HDL-c, LDL-c, and dipeptidyl peptidase-4 activity	[89,90,91]
Patients with ST-segment elevation MI	Alanine level elevation and lowering of the phospholipid content in very large HDL particles	--	Triglyceride levels in HDL and several HDL subfractions were measured 24 h post-MI; the composition of XS-VLDL (24 h post-MI) and L-LDL (baseline) was associated with abnormal LV function 4 months post-MI.	[92]
